# Research progresses in roles of LncRNA and its relationships with breast cancer

**DOI:** 10.1186/s12935-018-0674-0

**Published:** 2018-11-12

**Authors:** Xu Bin, Yang Hongjian, Zhang Xiping, Chen Bo, Yang Shifeng, Tang Binbin

**Affiliations:** 1 Department of Surgery, Zhejiang Rehabilitation Medical Center, Hangzhou, 310053 Zhejiang, China; 2 Department of Breast Surgery, Zhejiang Cancer Hospital, Banshanqiao, No. 38 Guangji Road, Hangzhou, 310022 Zhejiang China; 3 Department of Pathology, Zhejiang Cancer Hospital, Hangzhou, 310022 Zhejiang, China; 4 Second Outpatient Department of Traditional Chinese Internal Medicine, Tongde Hospital of Zhejiang Province, Hangzhou, 310012 Zhejiang, China

**Keywords:** LncRNA, Breast cancer, Promote, Inhibit, Target

## Abstract

Some progresses have been made in research of long non-coding RNA (hereunder referred to as LncRNA) related to breast cancer. Lots of data about LncRNA transcription concerning breast cancer have been obtained from large-scale omics research (e.g. transcriptomes and chips). Some LncRNAs would become indices for detecting breast cancer and judging its development and prognosis. LncRNAs may affect genesis and development of breast cancer in multiple ways. Perhaps they could develop into potential targets for treating breast cancer if they are carcinogenic. Like those from other studies of breast cancer, many data gained from omics research remain to be validated by much experimental work. For instance, it is still necessary to demonstrate reliability of LncRNAs as indices for diagnosing breast cancer and judging its prognosis (particularly for various subtypes of breast cancer), effectiveness and feasibility of these genes for treating breast cancer as targets. In this paper, recent years’ literatures about LncRNAs which are related to breast cancer are summarized and sorted out to review the research progresses in relationships between LncRNAs and breast cancer.

## Introduction

As a type of non-coding RNAs with over 200 nucleotides, LncRNAs may regulate physiological functions of organisms from the perspectives of epigenetics, transcription and post-transcription. Some of them are discovered to be involved in some important processes of breast cancer, including genesis, development, drug resistance and metastasis of breast cancer (BC), while some others may inhibit these processes. No matter they promote or inhibit the processes of breast cancer, their common mechanism of action consists in that they impact proliferation, apoptosis, drug resistance or invasion of BC cells. Some attempts are being made to develop some LncRNAs into targets for treating BC, and biomarkers for diagnosing BC, judging its prognosis or predicting metastasis and survival. In this paper, these years’ related literatures are collected, sorted out and summarized to review research progresses in relationships between LncRNAs and BC.

## A brief introduction to LncRNAs

Genomes of eukaryotes may transcript several types of RNAs, including protein-coding mRNAs, short and long non-coding RNAs (LncRNAs) [1]. From these RNAs, people have discovered that there are more non-coding RNAs than coding RNAs in human cells. According to encyclopedia of DNA elements (ENCODE), 76% human genomic DNA is transcribed into RNA [2]. The human genome project (HGP) indicates that only 2% genomic DNA is translated into protein [3], which reveals the existence of numerous non-coding RNAs. In these years’ research, short non-coding RNAs such as microRNAs (miRNAs), small interfering RNAs (siRNAs) and snoRNAs have been extensively studied. Meanwhile, LncRNAs are receiving growing concerns. More and more evidences have shown that LncRNAs are not simply considered to emerge as by-products of genomes, but have plenty of definite cellular functions, many of which are connected with human diseases.

LncRNAs are generally defined to be longer than 200 nucleotides and have no open reading frame that can be translated into protein. After analyzing genome tiling arrays and high-throughput sequencing of transcriptomes, numerous LncRNAs have been discovered. In these analyses, LncRNAs have been discovered to have complicated structures and origins, so researchers consider that they shall not be purely defined based on their length and non-coding. Research suggests that LncRNAs have some common characteristics as follows. (1) Coding LncRNAs are similar to genes of coding proteins in terms of chromatin states like H3K4me3 of promoters and H3K36me3 of transcribed regions [4]. (2) The expression of LncRNAs is regulated by multiple types of common transcription factors [5]. (3) Like coding genes, LncRNAs are transcribed with RNA polymerase II, generally spliced through spliceosomes and have poly A tails. According to the position of their DNA fragments in genomes, coding LncRNAs may be divided into five categories, including sense, antisense, bidirectional, intronic and intergenic LncRNAs. The position is more of less related to the functions of these genes.

In recent years, LncRNAs have drawn an increased attention because of their functions in the human diseases including cancers. They are involved in diverse biological processes such as cell proliferation, diferentiation, chromosome remodeling, epigenetic modulation, transcriptional and posttranscriptional modifcations [6, 7].

## Roles of LncRNAs

Some reports have claimed that more than 8000 types of LncRNAs have been discovered after a complete analysis of human genomes [8]. Some others have suggested that over 1000 types of LncRNAs are expressed in human beings and other mammals [4, 9]. In a word, there is a large amount of LncRNAs. However, the transcripts of several types of LncRNAs are not conservative among specimens with similar genetic relationships, and only over 200 types of LncRNAs have been investigated relatively clearly at present [10]. As a result, people question if all LncRNAs have biochemical functions, and to answer this question, further research shall be conducted.

The expression of most LncRNAs is tissue-specific. Among more than 200 types of LncRNAs which have been explored relatively clearly at present, many of them have been found to have functions in vitro, whereas only some of them have been demonstrated to function in vivo. It may be judged from current data that LncRNAs get involved in extensive biological and physiological processes, having distinct functions in different stages of these processes. Furthermore, LncRNAs, as crucial regulators for promoting or inhibiting tumor development, have been discovered to play their regulatory roles from the perspectives of epigenetic, transcriptional and post-transcriptional levels. In view of their roles in development of BC, LncRNAs are classified into two types: (1) some LncRNAs that promote the development of BC and (2) some others that inhibit the development of BC. No matter they promote or inhibit the development of BC, their mechanism of action generally covers several aspects as follows: (1) affect proliferation and apoptosis of BC cells, (2) influence drug resistance of BC cells, (3) impact invasion of BC cells.

## LncRNAs that promote development of breast cancer

**Fig. 1:**
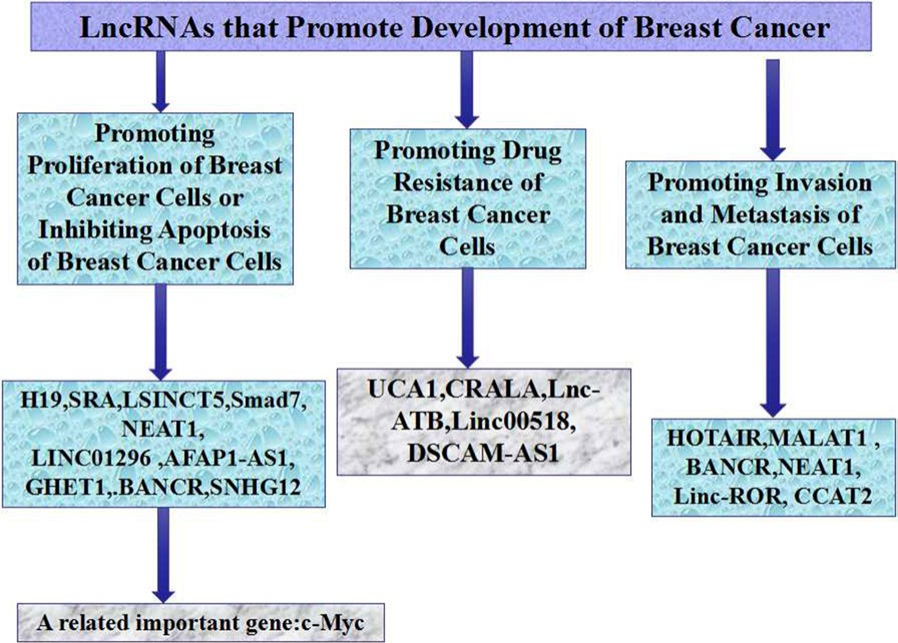
Related LncRNAs of promoting BC development

There is a range of LncRNAs that promote development of BC, and their functions have been preliminarily investigated. It is helpful for developing more effective means for diagnosing BC, judging its prognosis, predicting its genesis and intervening with the treatment. Hereunder, mechanisms related to these LncRNAs will be particularly introduced (see Fig. 1).

### Promoting proliferation of breast cancer cells or inhibiting apoptosis of breast cancer cells

#### H19

H19 is one of the first discovered LncRNAs, and located at the downstream of the sense of IGF2 in human genome. In multiple types of cancer, including BC, the expression of H19 is upregulated [11]. Compared with normal mammary tissues, the expression of H19 is higher in 72.5% of BC [12]. H19 may promote proliferation of BC cells when they are overexpressed in these cells. As a transcription factor, E2F1 may bind with promoters of H19 to enhance its expression and thereby lead to cell proliferation [13]. In addition, the overexpression of H19 in MDA-MB-231 contributes to the colony-forming efficiency and tumorigenic abilities in vivo [14]. In BC, the expression of H19 is higher in Estrogen receptor (ERα) positive cells, but in the ERα negative MDA-MB-231 cell line, ectopic overexpression of H19 is associated with increased proliferation [15]. It is clear that H19 is a category of LncRNAs that promote proliferation of BC cells.

#### Steroid receptor RNA activator (SRA)

Steroid receptor RNA activator (SRA) sites may be transcribed into encoded mRNAs or non-coding LncRNAs. It is the first LncRNA that was discovered to be not impacted by epigenetic regulation or catalytic regulation of enzymes. Selectively impacted by steroid receptors, it is a gene that gets involved in transactivating dependence of steroid receptors [16]. SRR is found to have higher expression in BC tissues and serum [17]. However, it has been discovered in animal experiments that pure excess SRA is inadequate for causing BC. This indicates that SRA promotes BC, but can’t lead to BC unless it acts together with other carcinogenic factors.

#### LSINCT5

As a type of about 2.6 kb long stress LncRNA, LSINCT5 is generally located in nucleus, and its genes are transcribed by RNA polymerase III instead of RNA polymerase II [18, 19]. LSINCT5 is overexpressed in several types of BC cells, so is it in BC tissues. The proliferation of BC cells will be reduced if LSINCT5 is knocked out of them [19]. It is thus clear that LSINCT5 is effective for increasing proliferation of BC cells, so it is deemed to promote the genesis of BC. It will be helpful for developing related reagents and drugs for diagnosing and intervening with BC by intensively exploring LSINCT5.

#### Smad7

As a type of genes located near Smad7 in mice, LncRNA-Smad7 has been reported to inhibit apoptosis of cancer cells with its expression in mammary epithelial cells and BC cell lines [20]. The anti-apoptotic functions of TGF-β may be mediated by suppressing the expression of LncRNA-Smad7. In contrast, ectopic expression of LncRNA-Smad7 may offset apoptosis induced by TGF-β receptor inhibitor. Nonetheless, LncRNA-Smad7 possibly only promotes the genesis of BC by affecting the apoptosis, because the knockout of its expression imposes no impacts upon TGF-β induced epithelial–mesenchymal transition, Smad2 phosphorylation or expression of Smad7. This indicates that LncRNA-Smad7 is a promoter of BC, but unlike other promoters, it merely inhibits apoptosis, so there would be new findings if it is further studied.

#### NEAT1

The human nuclear enriched abundant transcript 1 (NEAT1) gene encodes two LncRNA isoforms that play a central role in nuclear paraspeckles, which function in regulating RNA splicing and transcription. NEAT1 has been reported to play a critical role in mouse mammary gland development. NEAT1 functions as an oncogenic factor in multiple types of cancer, including BC, and its expression is under the regulation of ERαsignaling, the miR-449b-5p/c-Met axis, and hypoxia responses [21]. Studies have demonstrated that LncRNA NEAT1 promotes cancer progression. NEAT1 promoted invasion through inducing epithelial–mesenchymal transition (EMT) and NEAT1 played a role in 5-fluorouracil (5-FU) resistance. LncRNA NEAT1 could be a new diagnostic biomarker and therapy target for BC [22].

The meta-analysis by Yang et al. [23] suggest that there was significant difference in the OS between high NEAT1 expression level group and low NEAT1 expression level group. A significantly shorter OS was shown in the patients with high NEAT1 expression level than that with low NEAT1 expression level. Thus, it is implied that the increased expression level of NEAT1 was associated with poor OS. The meta-analysis results suggest the prognostic role NEAT1 in prognosis in the patients with different types of cancer. However, due to several limitations of the included studies, larger-sample size, multi-center and higher-quality studies with consistent criteria for defining high NEAT1 expression level and low NEAT1 expression level may be required to further confirm the current findings in this study. Above all, all aforementioned types of LncRNAs promote proliferation and inhibit apoptosis. It is favorable for understanding other similar LncRNAs by acquiring certain knowledge about these types of LncRNAs. Meanwhile, it will be helpful for developing pertinent means for diagnosing and intervening with BC by intensively investigating these genes.

#### LINC01296

LINC01296 exerts a tumor-promoting function in many cancers, in the regulation of proliferation and metastasis [24]. LINC01296 is up-regulated in both BC tissue samples and cells. Up-regulated LINC01296 is correlated with larger tumor size, positive lymph node metastasis, and advanced TNM stage of patients with BC. Additionally, Cox regression analysis confirmed LINC01296 as an independent prognostic indicator for patients with BC. LINC01296 may function as a potential prognostic predictor and therapeutic target for patients with BC [25].

#### AFAP1-AS1

LncRNA actin filament-associated protein 1 antisense RNA 1 (AFAP1-AS1) is a newly recognized cancer-related lncRNA deriving from the antisense strand of DNA at the AFAP1 coding gene locus. A slew of new studies suggest that AFAP1-AS1 is involved in many kinds of malignant tumors. Evidence has increasingly shown that AFAP1-AS1 could probably serve as a novel potential molecular biomarker in tumor diagnosis and therapeutic target in tumor treatment. A series of studies provide detailed information to understand lncRNA AFAP1-AS1 role in various human cancers. LncRNA AFAP1-AS1 is an oncogene in tumors that have been studied so far, and it may act as a useful tumor biomarker and therapeutic target [26]. The expression of AFAP1-AS1 was up-regulated in human BC tissue and associated with malignancy status, high expression of AFAP1-AS1 had a poor prognosis in BC patients. Up-regulated lncRNA AFAP1-AS1 indicates a poor prognosis in BC patients [27].

#### GHET1

Song et al.’s study for the first time revealed the biological functions of lncRNA gastric carcinoma highly expressed transcript 1 (GHET1) in BC. Their results demonstrated that GHET1 was up-regulated in BC tissues and cell lines, and promoted BC cell proliferation, invasion and migration by affecting EMT. Their research demonstrated that GHET1 was up-regulated in BC tissues and cell lines, and promoted BC cell proliferation, invasion and migration by affecting EMT [28].

#### BANCR

The role of the LncRNA BRAF-regulated LncRNA 1 (BANCR) in BC has not yet been elucidated. The present study revealed that BANCR was overexpressed in BC cell lines and tissues, and could promote the clinical progression of disease, including increases in tumor size, lymph node metastasis and tumor-node-metastasis stage. BANCR overexpression could promote the clinical progression, metastasis and proliferation of BC and indicate poor prognosis of patients with BC. BANCR may therefore be a potential prognostic marker and therapeutic target of patients with BC [29]. BRAF-activated non-protein coding RNA (BANCR) is a novel and potential regulator of cancer cell proliferation and migration [30]. LncRNA BANCR is highly expressed in BC, which is significantly correlated with the prognosis of patients. The down-regulation of BANCR can inhibit the proliferation, invasion and metastasis capacities of MCF-7 cells [31].

#### SNHG12

The long non-coding RNA (lncRNA) small nucleolar RNA host gene 12 (SNHG12) has a role in cell proliferation and migration. SNHG12 has been shown to play a role in a variety of human cancers [32]. The expression pattern of SNHG12 in BC and its clinical significance remains unclear. SNHG12 is upregulated in Triple-negative BC (TNBC), and its high expression is significantly correlated with tumor size and lymph node metastasis. Mechanistic investigations show that SNHG12 is a direct transcriptional target of c-MYC. SNHG12 contributes to the oncogenic potential of TNBC and may be a promising therapeutic target [33].

### A related important gene: c-Myc

Oncogenic c-Myc, which is located at chromosome 8q24, is one of the proto-oncogenic genes which is most frequently involved in human carcinogenesis [34]. Myc-induced transformation leads to the conversion from glucose to glutamine as the oxidizable substrate which is essential to maintain TCA cycle activity. c-Myc binds to the promoters and induces the expression of several crucial regulatory genes which are involved in glutaminolytic metabolism. It has been demonstrated that supra-physiological levels of Myc expression associated with oncogenic transformation are both necessary and sufficient for the induction of glutaminolysis to the excessive level that results in “glutamine addiction” specific to tumor cells.

The c-Myc mediates elevation of glutaminolysis in cancer cells. c-Myc promotes both glutamine uptake and glutamine catabolism. Because of c-Myc-mediated metabolic reprogramming, cancer cells tend to exhibit “glutamine addiction”. This is a typical example of metabolic reprogramming in cancer cells with oncogene-addiction, suggesting a potential “Achilles’ heel” of tumor cells that are addicted to glutamine metabolism in manner that is mediated by c-Myc [35]. The c-Myc/Max complex inhibits the ectopic differentiation of both types of artificial stem cells. Whereas c-Myc plays a fundamental role as a “double-edged sword” promoting both induced pluripotent stem cells generation and malignant transformation. Collectively, although the further research is warranted to develop the effective anti-tumor therapeutic strategy targeting Myc family, we should always catch up with the current advances in the complex functions of Myc family in highly-malignant and heterogeneous tumor cells to realize the precision medicine [36].

As a transcription factor, MYC’s primary mode of transformation is through the pro-tumorigenic transcriptional dysregulation of a wide variety of processes including proliferation, cell size, apoptosis, and metabolism. The alternative strategies of targeting MYC-driven cancers via selective inhibition of cellular pathways, like metabolism, that may selectively kill MYC-overexpressing cells have attractive therapeutic potential [37].

In a subset of neuroendocrine breast carcinoma tissues, the catalytic activity of EZH2 increases the number of the complex composed of N-Myc, AR, and EZH2-PRC2. Enhanced levels of EZH2 protein expression and EZH2 catalytic activity play a crucial role both in murine models overexpressing N-Myc and in human castration-resistant prostate cancer cells. N-Myc redirects EZH2 activity to N-Myc target gene promoters, resulting in the transcriptional suppression, whereas EZH2 inhibition reverses N-Myc-driven genetic regulation. Importantly, N-Myc sensitizes tumor cells to EZH2 inhibitors both in vitro and in vivo. In addition, N-Myc amplification rarely occurs in other lung cancer patho-histologic findings. N-Myc amplification also occurs in approximately 40% of neuroendocrine/small-cell prostate cancers, is commonly seen concurrently with amplification of the aurora kinase A gene (AURKA), and associated with poor prognosis.

Notably, downregulation of Myc interactor (NMI), a gatekeeper of epithelial phenotype, in breast tumors promotes mesenchymal, invasive and metastatic phenotype of the cancer cells. Aberrant miR-29 expression may account for reduced NMI expression in breast tumors and mesenchymal phenotype of cancer cells that promotes invasive growth. Reduction in NMI levels has a positive feedback machinery on miR-29 levels [36].

N-myc (MYCN), a member of the Myc family of basic-helix–loop–helix–zipper (bHLHZ) transcription factors, is a central regulator of many vital cellular processes. Overexpression of N-myc has been seen in a subset of BCs and correlates with poor prognostic features. Because phosphorylation of N-myc is directly regulated by Gsk3β, and indirectly by upstream signaling through the PI3K/Akt/mTOR pathway, targeting PI3K/Akt/mTOR may be an effective therapeutic approach [38]. NMI is an inducible protein whose expression is compromised in advanced stage breast cancer. Analysis of mRNA of NMI and miR-29 from patient derived breast cancer tumors showed a strong, inverse relationship between the expression of NMI and the miR-29. The related studies also revealed that in the absence of NMI, miR-29 expression is upregulated due to unrestricted Wnt/β-catenin signaling resulting from inactivation of GSK3β. Reduction in NMI levels has a feed-forward impact on miR-29 levels [39].

After neoadjuvant chemotherapy, 82.4% of patients showed pathologic partial response, with only 9.8% showing pathologic complete response. In multivariate analysis, MYC immunoreactivity and high MYC gain defined as MYC/nucleus ≥ 5 were significant predictor factors for pathologic partial response. MYC may have a role in chemosensitivity to AC and/or docetaxel drugs. The analysis of MYC amplification may help in the identification of patients that may have a better response to AC + T treatment [40].

### Promoting drug resistance of breast cancer cells

The growth of ER-positive BC is mostly dependent upon estrogen, so anti-estrogen therapies are major means for treating this type of BC in clinical practices. Nevertheless, these therapies can’t completely inhibit the growth of BC cells. BCAR4 (breast cancer antiestrogen resistance 4), as a type of LncRNA connected with resistance to tamoxifen which is an endocrine drug, may be detected in 10–27% BC samples. For patients with metastatic BC who are treated by tamoxifen, relatively high level of BCAR4 mRNA is associated with high degree of tumor malignancy and short survival. Certain report has suggested that in human BC cells ZR-75-1 and MCF7, cells can proliferate without estrogen and with various antiestrogen factors owing to artificial expression of BCAR4. Besides, BCAR4-induced resistance to endocrine drugs doesn’t depend upon estrogen receptors. Therefore, BCAR4 promotes BC by strengthening drug resistance of BC cells. As a type of highly transformed genes, it makes the growth of cancer cells not depend upon estrogen and drug-resistant to antiestrogen therapies. As a result, it contributes to the genesis of tumors in vivo. Meanwhile, some researchers consider that BCAR4-based high tumor-specific expression would be used for treating anti-estrogen BC as target. The research of Godinho et al. has suggested that both ERBB2 and ERBB3 are upregulated in the BC cell ZR that expresses BCAR4. In their research, the activation of ERBB2/ERBB3 signaling pathway is considered to be the reason why BCAR4 promotes drug resistance of BC. BCAR4-positive patients with BC would benefit from BCAR4-targeted therapies. Shi et al. [41] have discovered that Lnc-ATB is upregulated in BC cells and tissues which are resistant to trastuzumab (i.e. a type of monoclonal antibody for immunotherapies and drug for targeted therapies). It can be induced and activated by TGF-β. By competitively binding with miR-200c, it may promote the resistance to trastuzumab and induce invasion-metastasis cascade to upregulate expressions of ZEB1 and ANF-217. As a result, EMT is caused. In addition, researchers have discovered that high-level Lnc-ATB is connected with the resistance of BC to trastuzumab. These research findings imply that perhaps Lnc-ATP would cause EMT and resistance to trastuzumab in patients with BC as a downstream factor of TGF-β. Furthermore, Jiang et al. [42] have reported that LncRNA HIF1A-AS2 and AK124454 may not only stimulate proliferation and invasion of BC cells, but also play certain roles in promoting resistance of triple-negative BC to paclitaxel. Possibly used as signals for recurrence of BC, HIF1A-AS2 and AK124454 can be also utilized for strengthening therapeutic effects of paclitaxel as a target.

It is thus clear that some LncRNAs may increase resistance of BC cells to endocrine drugs and those for targeted therapies, so as to promote the genesis of BC.

#### UCA1

Recent studies reported that long non-coding RNAs (LncRNAs) might play critical roles in regulating endocrine resistance of BC. Urothelial carcinoma-associated 1 (UCA1) is an LncRNA with an oncogenic role in BC. Li et al.’s findings reveal that tamoxifen induces UCA1 upregulation in ER-positive BC cells in a HIF1α-dependent manner. UCA1 upregulation results in significantly enhanced tamoxifen resistance. miR-18a inhibitor reduced the sensitivity of MCF-7 cells to tamoxifen, while miR-18a mimics sensitized BT474 cells to tamoxifen. The upregulated UCA1 sponges miR-18a, which is a negative regulator of HIF1α. Therefore, UCA1 upregulation is further enhanced through a miR-18a-HIF1α feedback loop. In addition, our data also showed that miR-18a is a modulator of tamoxifen sensitivity due to its regulative effect on cell cycle proteins [43].

#### CRALA

The expression levels of chemoresistance-associated long non-coding RNA (CRALA), a newly discovered long non-coding RNA, CRALA is upregulated in chemoresistant BC cell lines. Silencing of CRALA in chemoresistant BC cells resensitizes the cells to chemotherapy in vitro. The univariate and multivariate analysis showed that higher CRALA expression was significantly associated with poor prognosis in BC patients. The study findings indicate that CRALA expression may be an important biomarker for predicting the clinical response to chemotherapy and prognosis in BC patients. It is possible to target CRALA to reverse chemoresistance in BC patients [44].

#### Lnc-ATB

Trastuzumab resistance (TR) is leading cause of mortality in Her-2-positive BCs, and the role of TGF-β-induced epithelial–mesenchymal transition (EMT) in trastuzumab resistance is well established, but the involvement of LncRNAs in trastuzumab resistance is still unknown. Shi et al. [41] identified long noncoding RNA activated by TGF-β (lnc-ATB) was the most remarkably upregulated LncRNA in TR SKBR-3 cells and the tissues of TR BC patients. They found that LncRNA-ATB, a mediator of TGF-β signaling, could predispose BC patients to EMT and trastuzumab resistance.

#### Linc00518

Linc00518 expression increased nearly twofold and MRP1 level elevated about 2.5-fold in BC tissues as compared to that in adjacent normal tissues. Linc00518 could act as a molecular sponge of miR-199a to repress MRP1 expression. MRP1 depletion increased the sensitivity of MCF-7/ADR cells to ADR, VCR and PTX, and this effect was attenuated following miR-199a inhibition or linc00518 overexpression. Also, linc00518 silencing increased ADR-mediated anti-tumor effect in vivo. linc00518 downregulation reduced MDR by regulating miR-199a/MRP1 axis in BC [45].

#### DSCAM-AS1

Ma et al. [46] investigate the influence of long noncoding RNA (LncRNA) DSCAM-AS1 on the propagation and apoptosis of tamoxifen-resistant (TR) BC cells via regulation of mircoRNA (miR)-137 and epidermal growth factor receptor pathway substrate 8 (EPS8). They think that LncRNA DSCAM-AS1 acts as a competing endogenous RNA of miR-137 and regulates EPS8 to promote cell reproduction and suppresses cell apoptosis in tamoxifen-resistant (TR) BC.

### Promoting invasion and metastasis of breast cancer cells

#### HOTAIR

Gupta et al. [47] have discovered that the expression of HOTAIR (i.e. a type of LncRNA) increases to different extent in primary BC tissues compared with normal mammary tissues, while it is upregulated by several hundred times and even nearly 2000 times in metastatic BC tissues. However, its expression is not so consistently high in primary tumors. In primary BC tissues, the high expression of HOTAIR may be considered as an index of cancer metastasis and patients’ survival [47, 48]. Additionally, highly expressed HOTAIR may enhance invasion of BC cells. Furthermore, high expression of HOTAIR has been discovered in in vivo experiments to more or less promote growth and spontaneous pulmonary metastasis of tumors. The knockout of HOTAIR may significantly decrease invasion of BC cells. In terms of its mechanism, it has been discovered that highly expressed HOTAIR induces site binding of PRC2 (i.e. polycomb repressive complex 2, a type of transcriptional inhibitor) and H3K27me3 (i.e. a kind of multiplex methylated histones) on 854 loci of genes, which are mostly downregulated by HOTAIR [47]. Chisholm et al. [49] have reported that in BC tissues, the expression of HOTAIR is positively correlated to EZH2, which is a subunit of PRC2. Compared with primary BC tissues, the expressions of both HOTAIR and EZH2 are significantly upregulated in metastatic BC tissues. Meanwhile, the co-expression of these two genes has positive correlations with poor prognosis of patients. The long noncoding RNA HOTAIR (HOX transcript antisense intergenic RNA) has been reported to be a biomarker for various malignant tumors; however, its involvement in BC is not fully understood. Han et al.’s study suggests the higher expressions of HOTAIR and EZH2 among three BC cells. Furthermore, the downregulation of HOTAIR or silencing of EZH2 was noted to inhibit the proliferation, invasion, and migration of BC cells, while promoting their apoptosis [50].

#### MALAT1

As a type of conservative LncRNAs, metastasis-associated lung adenocarcinoma transcript 1 (MALAT1) is highly expressed in multiple categories of cancer, including BC. The in vivo and in vitro experiments have demonstrated that MALAT1 may not only promote proliferation of triple negative BC cells, cancer development and metastasis, but also has negative correlations with the survival of patients with ER-negative HER-2 positive and triple negative BC in terms of its expression level [51]. In MCF7 (Luminal A) and MDA-MB-231 (triple negative), which belong to two kinds of cell lines, high-concentration 17b-Estradiol (E2) may not only inhibit cell proliferation, metastasis and invasion, but also reduce the level of MALAT1. The downregulation of MALAT1 may achieve similar effects, so E2 would impact cancer cells when the level of MALAT1 is decreased [52]. It has been discovered in research on MDA-MB-231 that KDM5B regulates MDA-MB-231 as a carcinogenic gene and the downregulation of KDM5B leads to lower expression of MALAT1 while decreasing invasion of cancer cells [53]. Above research indicates that MALAT1 has the potential to become an index for judging prognosis of BC and possibly turn into a target for treating BC.

The aforementioned studies suggest that both HOTAIR and MALAT1 may promote genesis and metastasis of BC by enhancing invasion of BC cells. Furthermore, MALAT1 may enhance proliferation of BC cells. Both LncRNAs are helpful for treating BC as targets for intervening with such cancer.

#### BANCR

The present study revealed that LncRNA BRAF-regulated LncRNA 1 (BANCR) was overexpressed in BC cell lines and tissues, and could promote the clinical progression of disease, including increases in tumor size, lymph node metastasis and tumor-node-metastasis stage. BANCR overexpression could romote the clinical progression, metastasis and proliferation of BC and indicate poor prognosis of patients with BC. BANCR may therefore be a potential prognostic marker and therapeutic target of patients with BC [29].

#### NEAT1

LncRNA NEAT1 was highly expressed in BC tissue, and the expression was also closely related to the tumor size and lymph node metastasis. Survival study also showed that the expression of lncRNA NEAT1 was closely related with prognosis of BC patients. LncRNA NEAT1 may act as an oncogene in BC, which can promote proliferation and metastasis of BC [54].

#### Linc-ROR

Hou et al. [55] have discovered that linc-ROR was upregulated in breast tumor samples, and ectopic overexpression of linc-ROR in immortalized human mammary epithelial cells induced an epithelial-to-mesenchymal transition (EMT) program. Moreover, they showed that linc-ROR enhanced BC cell migration and invasion, which was accompanied by generation of stem cell properties. Their results indicate that linc-ROR functions as an important regulator of EMT and can promote BC progression and metastasis through regulation of miRNAs. Potentially, the findings of the related study implicate the relevance of linc-ROR as a possible therapeutic target for aggressive and metastatic breast cancers.

#### CCAT2

The present study demonstrates that TAM-resistant cells show a higher level of long non-coding RNA CCAT2 expression compared with TAM-sensitive cells. Biologically, CCAT2 knockdown could inhibit proliferation and induce apoptosis in TAM-resistant cells exposed to TAM. Furthermore, knockdown of CCAT2 improves the sensitivity to TAM in TAM-resistant cells. Overall, the study results provide a novel therapeutic approach for TAM-resistant patients through depleting CCAT2 expression [56].

## LncRNAs that inhibit development of breast cancer

**Fig. 2:**
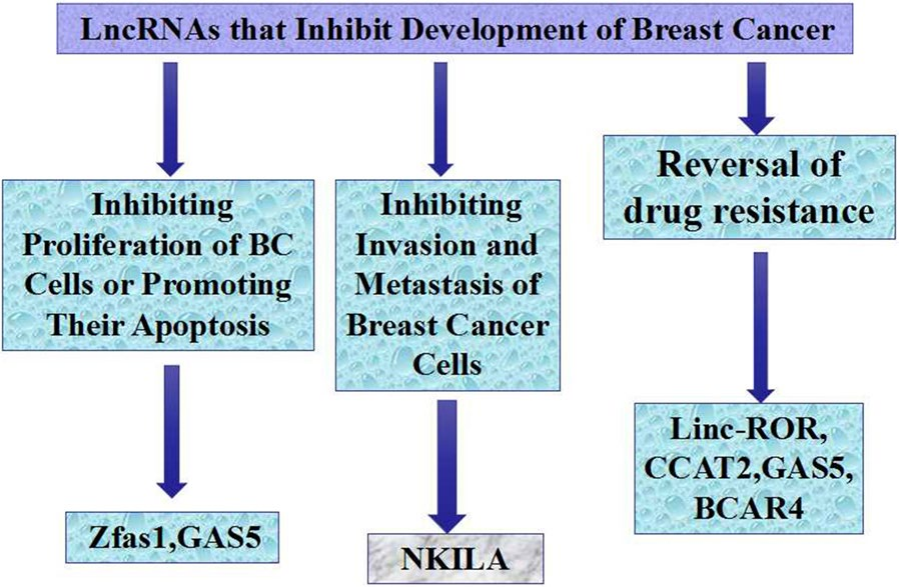
Related LncRNAs of inhibiting BC development

So far, some LncRNAs that inhibit BC from development have been explored relatively clearly. They have been discovered to mainly inhibit development of BC by inhibiting proliferation or stimulating apoptosis. See Fig. 2.

### Inhibiting proliferation of breast cancer cells or promoting their apoptosis

#### ZFAS1

Research has suggested that Zfas1, located inside mammary ducts and acinus, is expressed differently during pregnancy and breastfeeding, which has been found to be associated with breast development. After the knockout of Zfas1 from mammary epithelial cells, proliferation of cells is strengthened. Moreover, it has been found that the expression of Zfas1 is lower in BC tissues than that in normal breast tissues [57]. Hence, Zfas1 is a potential inhibitor of BC. Its functions and mechanism in the genesis of BC need to be further investigated, in hope of making certain contributions to developing means for intervening with and treating BC.

#### GAS5

GAS5 is another LncRNA that may cause cell growth arrest and apoptosis. Compared with peripheral normal cells, LncRNA GAS5 is significantly downregulated in BC cells. Mourtada-Maarabouni et al. [58] have reported that in MCF10A and MCF7 (BC cell lines), overexpressed GAS5 may increase the apoptosis rate of these cells under UV radiation and intervention of cisplatin as an anticancer drug. In line with above research findings, Ozgur et al. [59] have reported that in apoptotic MCF7 cells under genotoxic stress, the expression of GAS5 is upregulated. In some cases, the growth arrest and apoptosis of BC cell lines can be just caused by ectopic expression of GAS5. Besides, the expression of GAS5 is high in cells under growth arrest induced by lack of nutrients or growth factors, as a result of which these cells become extremely sensitive to the stimuli promoting apoptosis [60]. It is thus clear that some LncRNAs may inhibit BC by suppressing cell proliferation and stimulating apoptosis. These LncRNAs are expected to be developed and employed for intervening with BC.

### Inhibiting invasion and metastasis of breast cancer cells

Recently, Liu et al. [61] have reported a new LncRNA known as NF-KappaB interacting LncRNA (NKILA). Upregulated by NF-κB, it binds with NF-κB/IκB to produce stable composites, so as to directly cover the phosphorylated structural domains of IκB. As a consequence, IKK (IκB kinase) induced IκB phosphorylation and NF-κB are activated. Importantly, NKILA may prevent excessive activation of the NF-κB in mammary epithelial cells under inflammatory stimuli. In MDA-MB-231 (i.e. a BC cell line), NKILA may enhance apoptosis and reduce invasion. To sum up, NKILA may control invasion and metastasis of BC by inhibiting activation of NF-κB.

Thus, it may be inferred that some LncRNAs inhibit genesis and development of BC. In terms of its mechanism, it mainly plays its roles in inhibiting genesis and development of BC by suppressing proliferation of BC cells, or promoting apoptosis of these cells, or inhibiting invasion and metastasis of the cells. Nevertheless, no many LncRNAs have been discovered to be effective for inhibiting BC, and very few of them have been investigated from the perspective of their inhibition mechanism. It is hoped that researchers can discover more LncRNAs that can inhibit BC and examine them more clearly through their efforts.

### Reversal of drug resistance

Li et al.’s study explored the mechanism underlying long non-coding RNA ROR regulating autophagy on tamoxifen resistance in BC. These results indicate that inhibition of long non-coding RNA ROR reverses resistance to tamoxifen by inducing autophagy in BC [62]. The expression levels of chemoresistance-associated long non-coding RNA (CRALA), a newly discovered long non-coding RNA, were measured by quantitative real time-PCR in 79 pre-treatment biopsied primary BC samples. Small interfering RNAs were used to knockdown CRALA expression. The effect of CRALA on chemosensitivity was evaluated using cell growth assay. The study findings indicate that CRALA expression may be an important biomarker for predicting the clinical response to chemotherapy and prognosis in BC patients. It is possible to target CRALA to reverse chemoresistance in BC patients [44]. Gu et al. [63] investigates the role of LncRNA growth arrest-specific transcript 5 (GAS5) in tamoxifen resistance in BC. Their study demonstrates that GAS5 enhances the efficacy of tamoxifen in the treatment of BC and could be a novel prognostic biomarker.

BC antiestrogen resistance 4 (BCAR4) was identified in a functional screen for genes involved in tamoxifen resistance. BCAR4 is a strong transforming gene causing estrogen-independent growth and antiestrogen resistance, and induces tumor formation in vivo. Due to its restricted expression, BCAR4 may be a good target for treating antiestrogen-resistant BC [64]. BCAR4 may have clinical relevance for tumour aggressiveness and tamoxifen resistance. The research of Godinho et al. [65] suggests that BCAR4-positive breast tumours are driven by ERBB2/ERBB3 signalling. Patients with such tumours may benefit from ERBB-targeted therapy.

## Use of LncRNAs-related information

In spite of lack of complete and deep understanding about LncRNAs, people are not hindered from utilizing the conformed knowledge about these genes mainly for diagnosing BC, judging its prognosis and predicting its metastasis and survival.

### LncRNAs used as diagnostic markers

Ding et al. [66] have studied the possible applications of LncRNAs in diagnosing BC as a type of LncRNAs that exist among genes. They have found that lincRNA-BC2 and lincRNA-BC5 are generally upregulated by over twice in BC specimens compared with normal mammary tissues, whereas lincRNA-BC4 and lincRNA-BC5 are downregulated. For stage 3 BC, the expression of LincRNA-BC4 is significantly low, whereas the expression of lincRNA-BC5 is significantly high, and the expression of lincRNA-BC2 has significant positive correlations with LNM (lymph node metastasis). In particular, the expression of numerous LncRNAs is found to be highly associated with different molecular classification [67]. Furthermore, some research has suggested that LncRNAs in serum may be used for diagnosing genesis of BC. For instance, the expression of RP11-445H22.4 increases significantly in serum of patients with BC. The sensitivity and specificity for diagnosing BC with this are up to 92% and 74% respectively among Chinese people [68]. Liu et al. [69] aimed to develop a long noncoding RNA (LncRNA) expression signature that can predict response to tamoxifen. A set of LncRNAs (LINC01191, RP4-639F20.1 and CTC-429P9.3) associated with distant metastasis-free survival was established. Estrogen receptor-positive BC patients in the training series could be classified into high- and low-risk groups with significantly different distant metastasis-free survival values based on this signature. The LncRNA signature may have possible clinical implications in the selection of high-risk patients for tamoxifen therapy. It is thus clear that certain LncRNAs may be employed for diagnosing BC as biomarkers.

### LncRNAs used as prognostic biomarkers

Zhao et al. [70] have discovered a range of LncRNAs which may be used for differentiating patients with BC from low to high risks. The high expression of LINC00324 and low expressions of PTPRG-AS1 or SNHG17 are related to the relatively longer survival. Another study has suggested that SPRY4-IT1 promotes proliferation of BC cells by upregulating the expression of ZNF703. In addition, increased expression of SPRY4-IT1 is connected with higher tumor volume and more severe pathological staging in patients with BC [71]. Hence, SPRY4-IT1 is a type of new prognostic biomarkers and potential candidates of therapeutic targets.

The overexpression of HOTAIR in BC tissues is discovered to be connected with higher invasiveness and metastasis, which implies that HOTAIR would become biomarkers for predicting overall survival. The expression of HOTAIR may independently forecast if ER-positive BC is metastatic. MALAT1 is discovered to have higher expression in primary BC and its expression further increases in the course of metastasis [72]. On the contrary, the expression of BC040587 [73], NBAT1 [74] and EGOT [75] is discovered to be downregulated in BC tissues, which is associated with poor prognosis. Additionally, the high expression of LINC00472 in BC tissues is connected with poor invasiveness of BC cells and relatively good therapeutic effects [76]. Related clinical and in vivo studies suggest that LINC00472 is a type of tumor inhibitors, so it is possibly valuable for judging prognosis and predicting therapeutic effects of BC in clinical practices. This suggests that some LncRNAs may be used for judging prognosis of BC as biomarkers and have a great prospect for application in clinical practices.

MALAT1 is a highly conserved LncRNA that is highly expressed in several types of cancer, including BC. It has been demonstrated by in vivo and in vitro studies that MALAT1 promotes proliferation, tumor development and metastasis of TNBC. In addition, the expression of MALAT1 has been reported to be negatively correlated to the survival of ER negative, lymph node negative patients of the Her-2 and TNBC molecular subtypes. It is particularly noteworthy that a recent study using genetic interventions with MALAT1 antisense nucleotides has achieved good effects for suppressing cancer development in mouse models with luminal B BC. These studies suggested that MALAT1 is expected to become a new biomarker for prognosis of BCs and a potential target for treating them [77].

### LncRNAs used for predicting drug resistance and survival as markers

LncRNAs have potential value for being used for predicting drug resistance and survival of BC as markers. The overexpression of BCAR4 (a type of LncRNAs) is discovered to be effective for forecasting the resistance to tamoxifen which is a type of estrogenic drugs. On the other hand, LINC00160 and LINC01016 (i.e. two kinds of LinRNAs) are discovered to be highly expressed in ER-positive BC compared with ER-negative BC and normal tissues, which is discovered to have significant negative correlations with the overall survival of luminal A BC [78]. Additionally, a special BC subtype may be identified by detecting whether CCAT2 is overexpressed, having poor responses to adjuvant chemotherapies based on cyclophosphamide, methotrexate and CMF [79]. At last, the research of Chen et al. [80] has shown that the overexpression of ROR (i.e. a kind of LncRNAs) is correlated to chemotherapy resistance, which suggests that ROR may be used for predicting such resistance as an indicator. It may be inferred from the above that some LncRNAs are likely to become markers for predicting drug resistance and survival of BC.

Therefore, researchers are exploring the applications of LncRNAs from multiple perspectives (mainly including diagnosis of BC, judgment of prognosis, prediction of drug resistance and survival) based on their existing knowledge about them. Although it still needs some time to put them into practices, it is believed that the explorations of their applications will be more elaborate with the promotion of pertinent studies in terms of their breadth and depth. Meanwhile, new applications will be constantly developed.

## Conclusion

LncRNAs belong to a type of common transcription products in genomes of human beings and mammals. Since their relationships with the genesis and development of tumors have received growing attention, the functions and mechanisms of actions of some LncRNAs have been investigated clearly in preliminary research. Nevertheless, a majority of LncRNAs remain to be further widely and deeply studied. With the improvement of research methods like the development of gene array technologies and high-throughput sequencing technologies, more categories of LncRNAs are expected to be discovered. As people acquire a deeper understanding of LncRNAs, some of these genes are likely to be developed into biomarkers for diagnosing BC, judging and predicting its prognosis. Nevertheless, LncRNAs have been rarely reported to be used for intervening with BC. Recent CRISPR/CAS9 gene editing technologies would play certain roles in developing LncRNAs as therapies. For example, they can be employed for targeted silencing of certain LncRNAs that promote the genesis of BC.
